# Approaches for Benchmarking Single-Cell Gene Regulatory Network Methods

**DOI:** 10.1177/11779322241287120

**Published:** 2024-11-04

**Authors:** Karamveer, Yasin Uzun

**Affiliations:** 1 Department of Pediatrics, The Pennsylvania State University College of Medicine, Hershey, PA, USA; 2 Department of Biochemistry and Molecular Biology, The Pennsylvania State University College of Medicine, Hershey, PA, USA; 3 Penn State Cancer Institute, The Pennsylvania State University College of Medicine, Hershey, PA, USA

**Keywords:** Gene regulatory networks, single-cell genomics, epigenomics, benchmarking, ground truth

## Abstract

Gene regulatory networks are powerful tools for modeling genetic interactions that control the expression of genes driving cell differentiation, and single-cell sequencing offers a unique opportunity to build these networks with high-resolution genomic data. There are many proposed computational methods to build these networks using single-cell data, and different approaches are used to benchmark these methods. However, a comprehensive discussion specifically focusing on benchmarking approaches is missing. In this article, we lay the GRN terminology, present an overview of common gold-standard studies and data sets, and define the performance metrics for benchmarking network construction methodologies. We also point out the advantages and limitations of different benchmarking approaches, suggest alternative ground truth data sets that can be used for benchmarking, and specify additional considerations in this context.

## Key Points

- Single-cell sequencing data present new opportunities for constructing gene regulatory networks (GRNs).
- There are various performance measures for benchmarking GRN construction methods, including accuracy, stability, and scalability.
- The accuracy rates of constructed GRNs heavily depend on the selection of the performance metrics and ground truth networks.
- Ground truth data sets include regulatory databases, protein-protein interaction networks, and curated studies.
- Gene regulatory databases provide viable alternatives for specifically benchmarking transcriptional regulation.
- Protein interaction networks are generally used for benchmarking expression-based GRNs, although they often lack tissue specificity.

## Introduction

A gene regulatory network (GRN) is a set of directed regulatory interactions between gene pairs, in which a source gene (or protein) directly regulates the expression or function of the target gene (or protein). These models provide powerful analytical tools for comprehending the complex set of gene interactions that collectively drive cell differentiation and play critical roles in development and disease. Considering thousands of genes in the cell, the depiction of potentially millions of genetic interactions requires the use of efficient computational methods. Many different methods have been developed to computationally construct GRNs from genetic data, including correlation,^1^ mutual information,^2-5^ tree-based methods,^6^ regression techniques,^7,8^ Boolean networks,^9-12^ ordinary differential equations,^9,13-15^ neural networks,^16-18^ and Bayesian networks.^19-21^ A comprehensive review of GRN construction methodologies has already been provided elsewhere.^1,22-25^ GRN construction methods have been enhanced to take advantage of single-cell genomic sequencing data with an increasing number of published studies.^26-49^ These methodologies employ a diverse set of approaches, including repurposing bulk methods and novel approaches, as extensively discussed in the literature.^25,26,39,50-54^ Similar approaches have been used to construct GRN with single-cell multi-omics data,^55,56^ which are extensively discussed in recent review studies.^53,57^ In this article, we limit our focus to the benchmarking of GRN methods rather than the implemented methods themselves.

**Figure 1.**
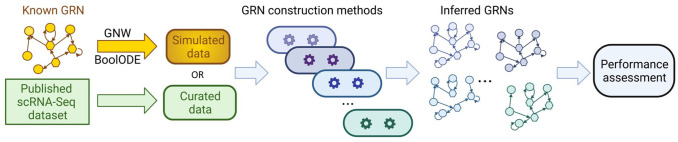
Common strategy for benchmarking GRN methods. The input data are generated by simulation software such as GeneNetWeaver or BoolODE using known networks or curated from published single-cell expression data. GRNs are constructed using state-of-the-art methods, which are then assessed for performance with different measures. GNW indicates GeneNetWeaver.

To correctly assess the performance of network methods, it is important and necessary to gain an understanding of the details of the gold-standard data sets and commonly used metrics. This general understanding will not only help investigators to choose an appropriate method that will satisfy their needs but will also contribute to reproducibility in this field by guiding the researchers who develop new methods to benchmark their own methods or perform new independent benchmarking studies. Many similar studies have been done earlier to compare network construction methods in different contexts.^58^ Although these benchmarking studies share a common framework (Figure 1), they differ in terms of selection of ground truth networks and performance metrics. In the rest of this article, we aim to present an overview of benchmarking strategies by defining the general gene network terminology, specifying the performance metrics, and describing commonly used ground truth data sets for benchmarking GRNs. We also present the strengths and limitations of each approach and provide additional suggestions for benchmarking.

In case of single-cell sequencing, the characteristics of this type of data, such as sparsity, noise, and different elements, can significantly impact the benchmarking of gene regulatory networks (GRNs).^39^ Dropouts occur when the expression of a gene is not detected due to technical limitations in scRNA-seq.^59^ This can introduce false negatives in the data, where true regulatory relationships might be missed because the expression of key regulators in certain cells is below detection thresholds. As a consequence of dropouts, many entries in the gene expression matrix can be zeros or very low values, leading to sparsity in the data. This can affect the accuracy of inferred regulatory relationships due to the fact that it can be hard to distinguish between a true absence of interaction and a technical artifact because of dropouts.^60^ This can lead to incomplete or inaccurate GRN inference. Single-cell data can also contain technical noise due to the sequencing process and biological noise from stochastic gene expression. Noise in the data can make it difficult to infer the true regulatory relationships between genes and can lead to false-positive or false-negative regulatory predictions. This, in turn, can affect the precision and recall of inferred GRNs.^61^ Therefore, methods that are robust to noise and incorporate noise models perform better in benchmarking studies. Due to the cellular heterogeneity, different cells in the same sample have distinct regulatory states and expression profiles, further complicating the identification of consistent regulatory interactions across cells. Benchmarking GRNs on heterogeneous data requires approaches that can handle variability and identify robust regulatory relationships across different cellular states. scRNA-seq expression data sets have narrow dynamic range due to the high proportion of genes having low expression levels.^62^ Therefore, these methods need to be sensitive enough to detect regulatory interactions even at low expression levels. Benchmarking studies need to evaluate the performance of methods across different expression ranges to ensure robustness.

## Classification of Gene Networks

Gene networks have been thoroughly studied through extensive literature, and the term GRN is used in different contexts with multiple meanings. For clarification purposes, we define a GRN as a set of *directed* regulatory interactions between gene pairs. These regulatory interactions are represented by edges originating from the upstream gene and destined to the downstream target gene that is being regulated. Based on this definition, there are 2 important characteristics of GRNs: (1) the set of nodes in a GRN consists of all types of genes and (2) all the edges between the genes are directed. The second characteristic draws a clear boundary between GRNs and another type of commonly used family of gene networks, namely, gene co-expression networks (GCNs).

**Figure 2.**
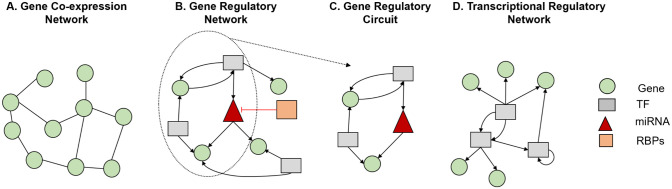
Different types of gene networks. (A) GCN: network with undirected edges. (B) GRN: network with directed edges. (C) GRC: a subnetwork of GRN that controls a biological function. (D) TRN: network with directed edges that can originate only from transcription factors (TFs).

Although GCNs also consist of interacting genes like GRNs, by contrast, the edges in the GCNs represent correlation relationships with no specific direction (Figure 2A and B). As the edges in a typical are undirected,^63^ it is not known which of the 2 connected genes is regulated by the other.^24^ It is worth noting that GCNs are distinct from GRNs in terms of their application in understanding biological systems and benchmarking of GCNs construction methods is provided in other studies.^64^ Hence, this review is focused on benchmarking strategies for GRN methods.

Another important network structure is gene regulatory circuits that focus on particular regulatory interactions within a biological pathway or process, compared with GRNs that offer a holistic perspective on the complete regulatory architecture within a cell or tissue.^65^ Gene regulatory circuits are akin to subcomponents of GRNs, delineating specific modules or functional units within the broader network (Figure 2C).

GRNs explain the regulatory processes governing gene expression. Causal networks and other gene association networks, however, provide useful information regarding statistical dependencies and correlations between genes, proteins, or other biological entities inside complex biological systems. These include GWAS/TWAS causal networks, which concentrate on determining the causal connections between genetic variations, alterations in gene expression, and phenotypic consequences.^66^ In terms of input, while causal networks from GWAS/TWAS integrate genetic variant and transcriptome data to infer causality, GRNs primarily use gene expression and regulatory data. In terms of output, GWAS/TWAS causal networks show the genetic foundations of various diseases and phenotypes^67^ whereas GRNs shed light on the dynamics and processes of cellular regulation.

The regulatory interactions in GRN can be executed by different types of regulators including transcription factors (TFs), microRNAs and RNA-binding proteins. Although often used interchangeably with GRNs, here, we distinguish the transcriptional regulatory networks (TRNs) as a specialized subcategory of GRNs that exclusively model gene expression control orchestrated by TFs. Although a TRN consists of all types of genes, the edges can only originate from genes that code for TF proteins, and they all represent transcriptional regulation (Figure 2D). We anticipate that this distinction will help clarify the terminology used in this field. In this context, most approaches for benchmarking GRNs can also be used for TRNs, as subsetting the originating nodes by selecting TFs only in a directed GRN yields a TRN. However, it should be noted that using a generic approach that discards the sequence specificity of TF binding events, can be less effective for benchmarking TRNs. This sequence specificity, which is defined as the distinctive affinity and selectivity of TFs for particular DNA sequences, known as binding motifs, is essential for the control of gene expression.

## Ground Truth GRNs

To benchmark GRN methodologies, a robust ground truth network is required to assess the accuracy of the inferred networks. A ground truth GRN can be constructed via genetic manipulation experiments such as genetic knockdown, knockout or overexpression. However, a full biological ground truth GRN can only be constructed via genetic manipulation (KO/OE) experiments with every single gene, individually. This is currently infeasible and the data to complete the full biological picture does not exist yet. Furthermore, ground truth data are even rarer for combinatorial effects. For TRNs, additional experimental epigenetic data, such as ChIP-Seq, may be used to unravel the epigenetic mechanisms of regulation.

Well-studied genetic organisms offer practical advantages for the assessment of genetic manipulations at scale, which helps to build GRNs using experimental techniques. Utilizing a unicellular model organism, such as *Escherichia coli* and *Saccharomyces cerevisiae*, can be a viable option for generating ground truth GRNs.^68^-^70^ Due to this practicality, the ground truth networks for these single-cell organisms are available in public repositories, such as DREAM (Dialogue on Reverse Engineering Assessment and Methods) network challenges,^1,71^ the RegulonDB,^72^ and other published studies.^73,74^ However, compared with multicellular eukaryotes, epigenetic regulation in these organisms presents important differences and has different roles. In multicellular eukaryotes, epigenetic regulation of gene expression is complex and controlled by promoters and enhancers, which are activated or repressed via complex molecular activation mechanisms, such as histone modification or CpG methylation. In addition to promoters and enhancers, regulatory DNA regions present in introns as well, which is unique to higher eukaryotes. Alternative splicing is another regulatory mechanism unique in multicellular eukaryotes to regulate the expression of different genes. Eukaryotes have extensive posttranscriptional regulation, including RNA processing (splicing, polyadenylation), RNA stability, and transport. In eukaryotes the same region of DNA can encode 2 different genes on opposite strands. By contrast, prokaryotes, such as *E. coli*, lack these mechanisms, and their gene regulation is primarily executed through operons.^75^ DNA methylation also influences the expression of bacterial genes, providing the ability of the bacteria to adapt to and survive in their environment and play a crucial role in phenotypic heterogeneity of bacterial populations.^76,77^ However, in bacteria, this mechanism is primarily executed via non-CpG methylation, which is different from methylation in eukaryotes. Moreover, prokaryotes have limited posttranscriptional regulation and their transcription and translation are often coupled. Although yeasts, such as *S. cerevisiae*, have histone proteins, their activation and repression mechanisms are highly different from multicellular eukaryotes, and they lack DNA methylation.^78,79^ Thus, these simple organisms may be underpowered to accurately model the gene regulation mechanisms in more complex organisms, such as mammals.

Alternatively, there are approximations of ground truth GRNs derived for specific multicellular eukaryote tissue or cell types, such as embryonic stem cells (ESC), hematopoietic stem cells, cortical area, cerebral cortex, spinal cord, reproductive cells, dendritic cells, hepatocytes, retina, and T-cells.^27,54,70,80,81^ These networks are obtained from different sources, including regulatory databases such as ESCAPE,^82^ a database that includes data sets^83,84^ from RNAi screens,^83,84^ IP-MS pull-down protein lists, differentially expressed genes following knockdown or overexpression, and target genes for TFs and histone modifications as identified by ChIP-seq; TRRUST^83,84^—a database generated by text mining from the published articles, contains human TF-target interactions with the mode of regulation, Rcistarget,^28^ list of binding sites for TFs identified via motif search; and RegulatoryCircuits,^85^ a GRN repository built on regulatory regions identified with CAGE^86^ experiments, motif analysis, and tissue-specific gene expression from human. Additional repositories include the Gene Transcription Regulation Database (GTRD),^87^ a database of TF binding sites (TFBSs) identified by ChIP-seq experiments for human and mouse; ChIP-Atlas,^88^ provides public data on ChIP-seq, ATAC-seq, DNase-seq, and Bisulfite-seq experiments; ChIPBase,^89^ a comprehensive transcriptional regulation atlas of ncRNAs and protein-coding genes (PCGs) built using ChiP-seq data. CistromeDB,^90^ a resource for human and mouse ChIP-seq, ATAC-seq, and DNase-seq data offers genome-wide maps of the locations of histone posttranslational modifications, chromatin remodelers, TFs, cofactors, and chromatin areas that are exposed to endonuclease activity. RegNetwork,^91^ a knowledge-based database of human and mouse GRNs is developed by compiling and combining the recorded regulatory interactions between TFs, microRNAs (miRNAs), and target genes from different databases. KnockTF^92^ contains extensive data sets of gene expression profiles in many tissues and cell types from various species, both before and after TF/TF-cofactors knockdown or knockout. NGS-QC^93^ is the largest collection of ChIP-seq and enrichment-related data sets collected from public databases. DoRothEA (Discriminant Regulon Expression Analysis)^94^ is a transcriptional regulatory database that can be used to search candidate TF-drug interactions and TF-Pharmacogenomic marker interactions in different cancers and reports the role of TFs in drug sensitivity across cancer cell lines screened with anti-cancer compounds (Table 1). These repositories provide tissue-specific networks for complex organisms inferred from histone modifications and accessible chromatin measured by epigenomic sequencing technologies. As a result, these databases can be valuable sources for benchmarking TRNs in the modeling of TF-based regulation.

**Table 1.** Gene regulatory databases.

Database	Organism(s)	URL
RegulonDB^72^	*E. coli*	regulondb.ccg.unam.mx
RegNetwork^91^	*Homo sapiens*, *Mus musculus*	regnetworkweb.org
TRRUST^84^	*Homo sapiens*, *Mus musculus*	grnpedia.org/trrust
ESCAPE^95^	*Mus musculus*	maayanlab.net/ESCAPE
GTRD^87^	*Homo sapiens*, *Mus musculus*	gtrd.biouml.org
ChIP-Atlas^88^	*Homo sapiens*, *Mus musculus, Rattus norvegicus, D. melanogaster, C. elegans, S. cerevisiae*	chip-atlas.org
ChIPBase^89^	*Homo sapiens*, *Mus musculus, Drosophila melanogaster*, *Caenorhabditis elegans*, *Arabidopsis thaliana*	rna.sysu.edu.cn/chipbase
CistromeDB^90^	*Homo sapiens*, *Mus musculus*	dc2.cistrome.org
KnockTF^92^	*Homo sapiens*, *Mus musculus*, *Arabidopsis thaliana* and *Zea mays*	bio.liclab.net/KnockTFv2
NGS-QC^93^	*Homo sapiens*, *Pan troglodytes*, *Gallus gallus, Mus musculus, Rattus norvegicus*, *Danio rerio*, *D. melanogaster, Arabidopsis thaliana, C. elegans, S. cerevisiae*	ngsqc.org
DoRothEA^94^	*Homo sapiens, Mus musculus*	https://saezlab.github.io/dorothea/
STRING^96^	*Homo sapiens, Rattus norvegicus, Mus musculus, Danio rerio, D. melanogaster, Arabidopsis thaliana, C. elegans, E. coli, S. cerevisiae, P. aeruginosa*, others inferred with orthology	string-db.org

Despite having the advantage of modeling sequence specificity, the regulatory databases have a major drawback. Although chromatin state experiments present valuable information about TF binding sites in DNA, they do not provide any information about the specific gene targeted by the TF. To determine the regulating TFs, these databases scan several thousand or tens of thousands of base pairs near the transcription start sites of the genes.^97^ Although practical to implement, scanning the flanking regions of transcription start sites for binding sites is not an accurate model of epigenetic regulation, as the enhancers to which TFs bind for gene regulation can interact with genes that are far more distant linearly (in terms of base pairs). An alternative approach can be to enlarge the scanning region for TF binding peaks.^98^ However, this would result in an ever-increasing number of regulating genes for a target gene parallel to the size of the region being used, thus reducing the specificity of the method. Hence, the sequence specificity and epigenomic data need to be complemented with the results of functional experiments. However, it should be noted that promoters are still the main regulators as enhancers cannot act alone.^99^

As gene regulation takes place via the proteins that are coded by the regulating genes, it can be modeled as the interaction of proteins, where 1 protein (TF) activates or inhibits the other (target). In this context, GRNs can be viewed as a subset of protein-protein interaction (PPI) networks and can be compared with PPI networks for assessing their accuracy. In this context, the STRING PPI database^96^ is used as a ground truth network for benchmarking GRNs.^68,80^ However, use of PPIs to benchmark GRNs have limitations and conceptual flaws. One limitation of using STRING is that it lacks tissue specificity. The STRING network is defined at the organism level, whereas GRNs act in a tissue-specific manner. However, future work in STRING is expected to include the option of pruning the edges based on gene expression information to provide tissue specificity.^96^ Another drawback is that as PPIs occur in different cellular locations, among very different biological processes and are not only found in the context of gene regulation. So, a TF which regulates the expression of a target gene doesn’t necessarily have to bind to the protein encoded by that target gene. In addition, the interactome of a TF can be very different from the interactome of another TF, but both TFs could regulate the same gene program. A simple example is that a TF can bind to a promoter of a gene, while another TF binds to an enhancer interacting with that gene. They both regulate the expression of this gene but the TFs do not interact physically with each other. Therefore, benchmarking with PPIs lacks biological justification and can produce misleading performance outputs due to a high number of false positives. Another problem with using STRING as the ground truth is that, as PPI databases merely capture protein interactions at a specific point, using them as ground truth networks depends on the strong assumption of correlation with the gene transcription. This assumption may fail to hold due to various intermediary levels such as mRNA degradation, translation, and posttranslational modifications.

Another important ground truth used in major studies is ChIP-seq data. Although ChIP-seq data are considered more accurate to infer GRNs, it has several limitations, primarily because TF binding does not definitively prove a regulatory relationship. ChIP-seq data identifies regions where TFs bind to DNA, but it does not directly demonstrate whether this binding can lead to gene activation or repression. TF binding can also occur without resulting in changes in gene expression, as binding alone does not guarantee functional regulation. Moreover, TF binding can be context-dependent. A TF may bind to different genomic regions under different cellular conditions or in different cell types. The understanding of the specific conditions under which TF binding occurs can be challenging. Furthermore, ChIP-Seq may not capture all regulatory interactions. It can only detect TF binding to accessible chromatin regions but can miss interactions where TFs bind to regions that are less accessible or where interactions are transient.^100^

Finally, curated networks, which are derived from individual genetic sequencing and manipulation studies, are also used as ground truth networks.^80^ Although these networks can be relatively accurate, they are often limited in terms of network size. Hence, although they can be useful as simplistic models, they may be insufficient for general purpose benchmarking.

Overall, the available regulatory databases and repositories offer the ability to reflect the regulatory mechanisms of complex organisms, such as multicellular eukaryotes. However, their representative power may be limited, as some of the interactions in these repositories may lack genetic manipulations. For the repositories having genetic manipulation data, the modeling accuracy of such manipulations may be restricted due to the reduced ability of control for the experimental conditions in complex organisms. Presumably, if 1 protein (TF) activates or inhibits the other (target), the target can also be a TF. However, it may also be possible for RNA-binding proteins (RBPs) to interact with each other, or with TFs/cofactors. These other interactions (Table 2) can be useful in construction of a complete regulatory interaction network.

**Table 2.** Other interaction databases.

Type of interaction	Database	URL
TF-miRNA	TransmiR v2.0^101^ TMREC^102^ CircuitsDB^103^ TRmir^104^	http://www.cuilab.cn/transmir http://bioinfo.hrbmu.edu.cn/TMREC/ http://biocluster.di.unito.it/circuits/ http://bio.liclab.net/trmir/index.html
RBP-RNA	RBPDB^105^ RNAct^106^ CLIPdb^107^ EuRBPDB^108^	http://rbpdb.ccbr.utoronto.ca https://rnact.tartaglialab.com/ http://clipdb.ncrnalab.org/ http://eurbpdb.gzsys.org.cn/
Transcription cofactors and transcription factor interacting proteins	TcoF-DB^109^	http://tcofdb.org/

The applicability of these ground truths can vary on types of interactions in the inferred network. Most physical interaction databases such as PPIs do not provide directionality information for the regulations. However, other databases such as regulonDB^72^ and KnockTF^92^ can provide the directionality as well as the mode (activation/suppression) information for the curated regulatory interactions.

## Performance Metrics

**Figure 3.**
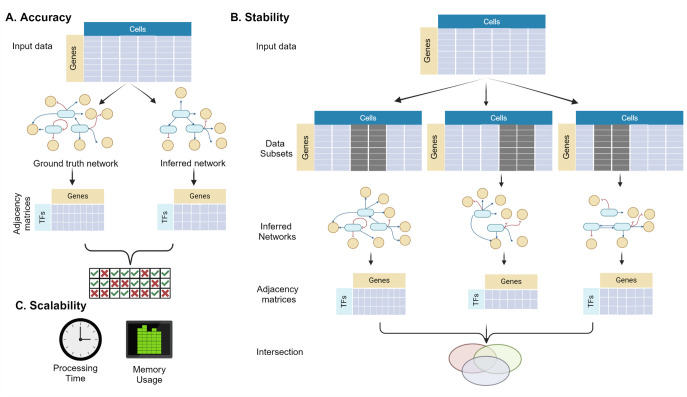
Performance measures for evaluating GRN construction methodologies as a whole. (A) Accuracy measures how consistent the inferred network is with the ground truth, whereas (B) stability reflects consistency across different inputs for the same tissue/cell type. Cells with gray background denote the cells excluded from the analysis. (C) Scalability shows the computational efficiency of a method in constructing a network in terms of time and memory requirements.

When evaluating GRN construction methods, different aspects of performance need to be considered. Although accuracy is often considered the most important performance measure since it reflects the consistency of the network with cellular biology; other measures are also critical for assessing the usability of the method. These performance measures include (1) accuracy of the predicted network topologies when compared with ground truth networks, (2) stability as a measure of robustness and reliability of the algorithm, and (3) scalability of the algorithm in terms of computational resource needs (Figure 3). Accuracy is determined by comparing the generated network topology with a selected ground truth GRN. Stability, by contrast, measures reproducibility by comparing the output of a GRN method by itself with different inputs for the same condition.^81^ It can be computed by comparing multiple GRNs generated with various inputs for the same condition, which may correspond to different data sets, replicates, or bootstrapped samplings from the same data set.^56^

**Figure 4.**
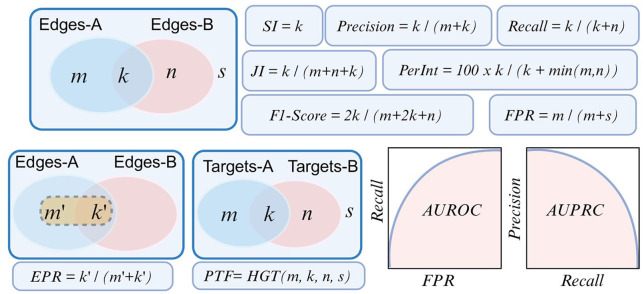
Performance metrics for determining the accuracy and stability of the constructed GRN networks. Orange shaded set on the left of the bottom row represents the predefined number of top-ranking interactions in set A. AUPRC indicates area under the precision-recall curve; AUROC, area under the receiver operating characteristics curve; EPR, early precision rate; FPR, false positive rate; F-score, harmonic mean; HGT, hypergeometric test; JI, Jaccard index; PerInt, percentage of intersection; PTF, predictable transcription factors; SI, set intersection; U, universal set; WJS, weighted Jaccard similarity.

A variety of metrics are used for comparing the 2 networks to assess accuracy and stability (Figure 4 and Table 3). The simplest network comparison measure is the set interaction (SI), in which the individual edges between the 2 networks are compared in a pairwise manner as in equation (1), where A represents the set of inferred edges and B is the set of ground-truth interactions.^68^ The size of the overlap between 2 sets provides a measure of consistency. Despite its simplicity, this metric can be severely affected by the size of the networks under comparison. A predicted network with a very large size will naturally have a large intersection with any set, leading to unwanted bias

(1) SI=|A∩B|

**Table 3.** Metrics of performance for assessing GRN construction methodologies.

Study	Accuracy	Stability	Scalability
Chen and Mar^68^	AUROC, AUPR, SI	NA	NA
Pratapa et al^80^	AUROC, AUPR, EPR	JI	Run time, memory
Nguyen et al^54^	AUROC	NA	Run time
Zhao et al^69^	AUPR, AUROC	AUPR, AUROC	NA
Kang et al^81^	Precision	PerInt, WJS	NA
McCalla et al^70^	AUPR, PTF, F-score, JI	NA	Run time, memory

Abbreviations: AUPR, area under the precision-recall curve; AUROC, area under the receiver operating characteristics curve; EPR, early precision rate; JI, Jaccard index; PerInt, percentage of intersection; PTF, predictable transcription factors; SI, set intersection; WJS, weighted Jaccard similarity.

Jaccard index (JI) attempts to address this issue by normalizing the intersection size by the size of the set union as in equation (2).^70,80^ Precision and recall metrics normalize the size of the set intersection with the number of edges in the predicted network and the ground-truth network, respectively.^81^ As an alternative normalizing factor, the percentage of intersection (PerInt) uses the minimum of the sizes of the 2 sets as in equation (3).^81^ Similarly, the F1-score measures network conformation as a harmonic mean of precision and recall as a more balanced metric as in equation (4).^70^ In fact, F1-score is a specialized form of F-beta score, which also allows one to decide the weight for the balance between precision and recall using the beta parameter. Different beta values for the F-beta score helps to tailor the metric for specific tasks. Beta values less than 1 emphasizes minimizing false positives rather than minimizing false negatives, while beta values larger than 1 works in the opposite way. In GRN performance evaluation, both precision and recall are critically important and the F1-score typically provides a good compromise between the 2 and therefore is more commonly used for GRN accuracy evaluation than F-beta score (equation (5)).

All these metrics are based on binary comparisons of edges across the networks. However, the predictable TF (PTF) metric is different in the sense that it compares the targets of the TFs across 2 networks,^70^ providing a set of values corresponding to different TFs. It uses a hypergeometric test to determine the significance of the intersection of inferred interaction with each interaction in the ground-truth network. Hypergeometric test employs the hypergeometric distribution to determine the statistical significance of selecting a sample of a given number of k successes (out of n total draws) from a population of size N that contains K successes

(2) $$\mathrm{JI}=\frac{\left|A\cap B\right|}{\left|A\cup B\right|}$$

(3) $$\mathrm{PerInt}=100\times \frac{\left|A\cap B\right|}{\min\left(\left|A\right|,\left|B\right|\right)}$$

(4) $$\mathrm{F1}-\mathrm{score}=\frac{\left|A\cap B\right|\times 2}{\left|A\cap B\right|+\left|A\cup B\right|}$$

(5) $$F-\mathrm{beta}\;\mathrm{score}=\frac{\left(\left(1+{\mathrm{beta}}^{2}\right)\;*\;\mathrm{Precision}\;*\;\mathrm{Recall}\right)}{{\mathrm{beta}}^{2}\;*\;\mathrm{Precision}+\mathrm{Recall}}$$

The metrics described above assume that the predicted network is already pruned and discards the weights of the edges. However, some GRN methodologies do not prune the output network but assign weights to the edges on a continuous scale. For such cases, alternative numeric metrics are used for comparison. One such metric is the early precision rate (EPR), which compares the predefined number of top predicted edges with the ground-truth network.^80^ This metric is easy to compute and interpret, but there is no common rule for the selection of the number of top edges, making this choice of parameter arbitrary, which can potentially affect the outcome.

Accuracy is an important metric, however when dealing with binary classifiers it can be biased due to the imbalanced nature of single-cell data. Therefore, it is important to select the evaluation metrics that can minimize the effect of sparsity, data imbalance, and can improve performance measurement. Recent studies proposed new metrics for correctly evaluating inferred GRNs such as balanced accuracy, area under the receiver operating characteristics curve (AUROC) and area under the precision-recall curve (AUPRC).^110^ Balanced accuracy adjusts the accuracy by accounting the number of positive and negative instances to handle imbalanced data sets by averaging the accuracy of each class. Precision-recall (PR) curves eliminate the necessity of parameter choice by computing the precision (equation (6)) and recall metrics (equation (7)), by moving the cutoff on a continuous scale.^111^ Here, accuracy is defined by the AUPRC as a metric that is agnostic to the selection of a cutoff for network comparison.^68-70,80^ However, AUROC and AUPRC might be biased for imbalanced data sets. Another drawback is that the absolute value of the AUPRC does not directly suggest a direct interpretation of success when compared with a random guess. This arises from the fact that there is not an intuitive value for expected area under the curve (AUC) and it depends on the composition of positive and negative examples (edge labels) on the data. Hence, either the expected AUC needs to be stated in the evaluation or the performance of a random predictor needs to be shown with a separate curve as a baseline comparison

(6) $$\mathrm{Precision}=\frac{\left|A\cap B\right|}{\left|A\right|}$$

(7) $$\mathrm{Recall}=\frac{\left|A\cap B\right|}{\left|B\right|}$$

Receiver operating characteristics (ROC) curves show the recall values against false positive rate (FPR) on a continuous scale with a moving threshold as in equation (8), where U represents the universal set. Intuitively, this metric provides a comparison with the random guess as the baseline.^112^ In a typical ROC curve for a balanced data set, the 45° (hypotenuse) line theoretically approximates the outcome of random guessing with an AUC value of 0.5. Comparing individual methods to this baseline offers an important advantage by showing whether any proposed method can provide any meaningful information. We find this property particularly important because the AUROC values reported in independent benchmarking studies^54,68,69^ are mostly in the proximity of 0.5. Thus, despite the existence of a large number of published GRN construction methods, only a small number of them are able to provide AUROC values that are meaningfully higher than 0.5, which is a crucial insight that can only be provided through ROC curves. Weighted Jaccard Similarity (WJS) is a metric which also takes into account the similarity of the weights associated with the shared links between the 2 compared networks as in equation (9)

(8) $$\mathrm{FPR}=\frac{\left|A\backslash B\right|}{\left|U\backslash B\right|}$$

(9) $$\mathrm{WJS}\left(A,B\right)=\frac{\Sigma_{i=1}^{\left|N\right|}\;\min\left(w_{i}^{A},w_{j}^{B}\right)}{\Sigma_{i=1}^{\left|N\right|}\;\max\left(w_{i}^{A},w_{j}^{B}\right)}$$

where w^A^ and w^B^ are the vectors of weights associated with the common links (N) between A and B.

Cross-validation is another approach that has been used to evaluate the performance of GRN inference methods, particularly those based on regression on gene expression and especially when no good ground truth exists. This technique divides data into subsets and measures the performance of methods on unseen data sets. Cross-validation helps in assessing how well the GRN model generalizes to unseen data and avoids overfitting by providing a more realistic estimate of its performance.^113^ It is a crucial step in the evaluation and validation of GRNs to ensure robustness and reliability in regression based GRN construction methods. However, reliability of this approach mainly depends on the sampling strategy. Random cross-validation (RCV) can be influenced by an overoptimistic performance evaluation; therefore, the model may not be generalized to new biological contexts. This is because RCV may conflate samples that are seen and samples that are not seen by placing samples with high similarity in both the training and test sets. A repeated stratified random sampling based cross-validation approach can be a good alternative for small sample sizes. Clustering-based cross-validation is used for more realistic estimation of error on distinct test samples. However, the choice of clustering method majorly affects this evaluation. This evaluation depends on selection of the clustering algorithm and its parameters such as number of clusters and initialization. In this case, the distinct sets (the clusters) may be at different distances from each other and averaging the estimated performance over all clusters may not be a reliable estimate of performance on new samples.^114^ Therefore, a clustering method independent approach such as simulated annealing (SA), is used that allows to construct test/training partitions with required degrees of distinctness, which is a measure that quantifies how similar a test set and a training set are to each other.^113,115,116^ In this context, the fitness function in the SA method measures how distinct the test set is from the training set. This function would guide the SA algorithm to iteratively improve the partitions, making the test sets more challenging by increasing their distinctness. The fitness function is critical because it directly influences how the partitions are optimized.

Evaluating models in biological research, particularly GRNs, presents hurdles because of the inherent sparsity of biological systems and the absence of known true negatives. It is important to select the evaluation metrics that can minimize the effect of sparsity and can improve performance measurement. Precision-Recall, F1-score, and positive predictive value (PPV) can handle the natural sparsity in single-cell GRN-construction. In our view, true positives are more important than true negatives since true positive reflects real regulatory interactions that can be used for further downstream analyses. In the same manner, minimizing false positives is more important than false negatives as it is highly critical for the reliability of predicted interactions. These metrics majorly focus on the true positives in predictions. To address the lack of known true negatives metrics such as Precision at top-k, AUPR, and ranked-based metrics (mean average precision and normalized discounted cumulative gain) perform better. These metrics are majorly based on ranking the true positive predictions and using the predictions with high rank in evaluation. Moreover, the evaluation of methods based on the information in the output network such as directionality and sign of regulation (activation/suppression) requires specific metrics. Precision-Recall, F1-score, AUPRC, and AUROC can handle diverse types of networks such as directed signed, directed unsigned, and undirected networks whereas metrics such as JI and PerInt cannot use the directionality and sign information and therefore have limited usage in undirected networks (Table 4).

**Table 4.** Commonly used metrics for evaluating gene regulatory networks and the characteristics of networks that they can be used to evaluate.

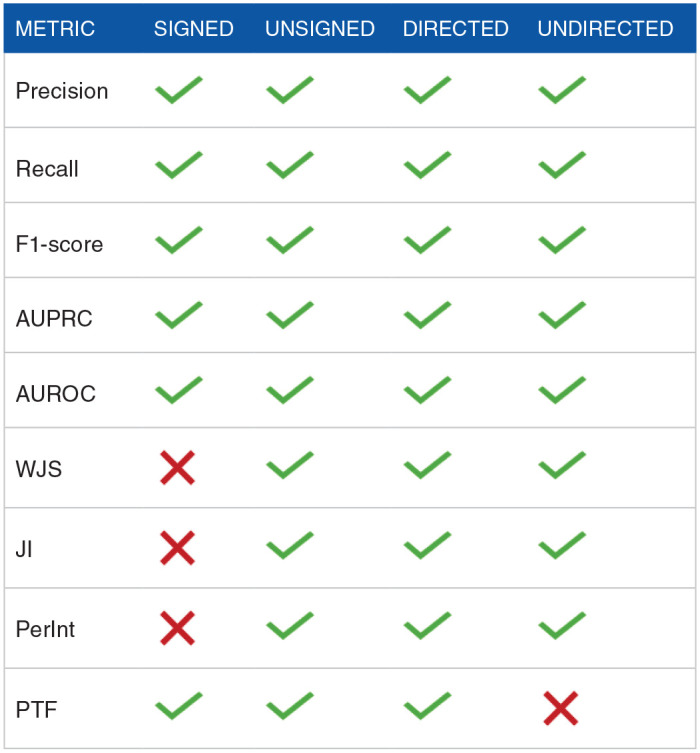

In addition to measuring accuracy and stability using a chosen metric, it is imperative to assess the computational scalability of a GRN method. The demonstration of the performance of a newly developed GRN method on a small-scale data set with several genes is often insufficient for general purpose benchmarking. Hence, it is necessary to measure how scalable the algorithm is and how successfully it can infer a network where there are thousands of genes as the input, as in the case of most research problems. Scalability is assessed by recording the processor usage time and maximum memory allocation to infer the network with varying input sizes (Figure 3C). Although hardware resources are expanding rapidly, an algorithm that is not well-scaled can require exponentially increasing resources to complete, resulting in depleted resources and possibly failing to generate an output, limiting their potential usage.^54,70,80^ Hence, it is critical to evaluate the scalability of benchmarking GRN methodologies in terms of computational resource requirements.

Different model types have varying capacities to scale with data set size or complexity of biological interactions. Machine learning based models such as Random Forests (GENIE3^6^ and GRNBOOST2^117^) and support vector machines are scalable for moderate data size (~2000 genes).^80^ Statistical models such as linear regression, logistic regression, or generalized linear models (GLMs) are considered to be scalable for large data sets. However, pseudotime requiring methods based on mutual information (SCRIBE^118^ and PIDC^39^), and regression models (SINGE,^119^ GRISLI,^120^ and GRNVBEM^37^) are not scalable enough on a data set with more than ~4000 genes. Boolean models (SCNS^121^ and BTR^122^) tend to have more scalability issues as the number of combinations grows exponentially with the number of inputs.^70^ However, to deal with scalability issues, recently developed high performance computation techniques such as Cartesian genetic programming are being used on GPU.^123,124^

## Benchmarking Studies

The benchmarking studies help to facilitate fair comparisons between different GRN inference methods and their reproducibility. GRNs are complex systems with numerous interactions between genes, proteins, and other molecules; accurately modeling their behavior is a significant challenge in computational biology. Therefore, the available GRN benchmarking studies employ different strategies, gold-standard networks, and performance metrics as described above.^54,68-70,80,81^ Some studies have used simulated data as input data sets using GeneNetWeaver^125^ or BoolODE,^80^ while others have used data sets curated from published studies. Studies also differ in terms of methods used, such as pseudotime calculation-based and time-independent methods. The regulatory databases, protein interaction repositories, networks from the DREAM challenges, and curated networks from literature have been used as ground truth data sets to evaluate accuracy (Table 5).

**Table 5.** The gold-standard data sets generated and used for independent benchmarking studies.

Study	Network type	Organism(s)	Cell type(s)	Gene count	Input	Ground truth network(s)
Nguyen et al^54^	GRN	*Homo sapiens*	Multiple	20-3000	Sim.-GNW^125^	TRRUST^83,84^
Chen and Mar^68^	GRN	*E. coli*	PC	100	Sim.-GNW^125^	RegulonDB^72^
		*E. coli*	PC	10		
		*Mus musculus*	HSC	33	Curated^29^	STRING^96^
		*Mus musculus*	ESC	96	Curated^126^	
Pratapa et al^80^	TRN	*Mus musculus*	CC	5	Sim.-BoolODE^80^	Curated^127^ and Synthetic networks^128^
		*Homo sapiens*	VSC	8		Curated^129^
		*Homo sapiens*	HSC	11		Curated^130^
		*Homo sapiens*	SPC, GPC	19		Curated^131^
		*Mus musculus*	HSC-E	656	Curated^132^	STRING^96^, ESCAPE^82^ (only for mouse ESC), Cell-type specific ChIP-seq^82,133,134^, non-specific ChIP-seq^84,91,94^
		*Mus musculus*	HSC-L	539		
		*Mus musculus*	HSC-GM	592		
		*Mus musculus*	ESC	995	Curated^135^	
		*Mus musculus*	DC	764	Curated^136^	
		*Homo sapiens*	ESC	843	Curated^137^	
		*Homo sapiens*	Hep.	909	Curated^138^	
Zhao et al^69^	TRN	*E. coli, S. cerevisiae*	SCO	10 100	Sim.-GNW^125^	DREAM4^71^
		*E. coli*	PC	9	Curated^139,140^	SOS DNA Repair Network^139,140^
		*E. coli*	PC	1484	Curated^141^	DREAM5 ^1^ + RegulonDB^72^
Kang et al^81^	TRN	*Homo sapiens*	Retina	6212	Curated^142,143^	Rcistarget^28^, RegulatoryCircuits^85^
			T-cells	11 242	Curated^144,145^	
			HSC	7038	Curated^146,147^	
McCalla et al^70^	TRN	*Homo sapiens*	ESC	12 986	Curated^148^	Curated^148^
		*Mus musculus*	ESC	6156	Curated^149,150^	Curated^95,151-156^
		*Mus musculus*	DC	9394	Curated^136^	Curated^136^
		*S. cerevisiae*	SCO	2876	Curated^73,74^	Curated^73,74^

Abbreviations: CC, cerebral cortex; Curated, real scRNA-seq data curated from published studies; DC, dendritic cells; -E, erythroid differentiation; ESC, embryonic stem cells; GM, granulocyte-macrophage differentiation; GNW, GeneNetWeaver; GPC, granulosa progenitor cells; Hep, hepatocyte-like cells; HSC, hematopoietic stem cells; -L, lymphoid differentiation; NA, not applicable; PC, prokaryotic cell; SCO, Single-celled organism; Sim, scRNA-seq data that is generated via simulation software; SPC, Sertoli progenitor cells; VSC, ventral spinal cord.

Due to the advantage of being fast, easily reproducible, and less expensive than biological experiments, the synthetic data generated from GeneNetWeaver is used in many studies. However, it is designed for bulk RNA-seq data and cannot handle the noise in single-cell data. The addition of (usually Gaussian) noise in this method does not tend to correctly represent the dynamics of gene regulation when dealing with single-cell data.^64^ Moreover, it has limitations for simulating pseudotime based data. Recently, GRN-guided simulator based methods such as BoolODE,^80^ SERGIO,^157^ and GRouNdGAN^158^ have been proposed as better alternatives to generate synthetic data sets. BoolODE is specifically useful in benchmarking pseudotime requiring methods, as it can generate diverse network topologies such as linear, bifurcating, trifurcating structures. It uses a similar model to the one used in GeneNetWeaver with the exception that the regulators of each gene are not combined using a random logic function. Instead, it uses the known Boolean function that specifies how the states of the regulators control the state of their target genes. Another difference is that BoolODE independently samples each parameter for every data set from a normal distribution using the value as the mean and a standard deviation of up to 10% of this mean value. This allows BoolODE to add information about the real interactions instead of random guesses. SERGIO, a simulator of single-cell gene expression data, also uses stochastic models based on a user-provided gene regulatory network. SERGIO works with any number of cell types, steady state or cells differentiating to multiple fates. The data sets generated are statistically comparable with experimental data generated with different platforms such as Illumina HiSeq2000, Drop-seq, Illumina 10X chromium, and Smart-seq.^157^ Similarly, GRouNdGAN (GRN-guided in silico simulation of single-cell RNA-seq data using Causal generative adversarial networks) is a deep learning based causal implicit generative model. It is built for reference-based GRN-guided simulation of scRNA-seq data. GRouNdGAN learns the co-regulatory patterns through complex functions instead of simplifying assumptions and elaborate regulatory dynamics.^158^ The causal structure of GRouNdGAN makes it useful for performing in silico knockout experiments. For the methods which require time information/cell ordering, dynverse^128^ provides diverse network topologies as used in BEELINE.^80^ Recently some methods have been developed to generate synthetic data set for multiple modalities required for GRN construction. scDesign3^159^ is designed to generate realistic in silico data with negative and positive controls for multimodal single-cell and spatial omics experiments.^159^ scMultiSim^160^ is another simulator for multi-modality single-cell data generation; it is guided by cell-cell interactions and GRNs.^160^ It also incorporates noise and batch corrections; however, it requires the predefined cell trajectories which may not be available in many cases. To assess their true applicability and accuracy, new benchmarking studies are required on diverse multi-omics based GRN inference methods.

As one of the early benchmarking studies in this context, Chen et al^68^ compared the performance of 5 general and 3 single-cell specific gene regulatory network reconstruction methods on experimental and synthesized single-cell data. The experimentally generated data sets (ESC and hematopoietic stem cells [HSC]) for mouse were used in this study and the interactions in the STRING database^161^ used as the ground truth for this data. In addition, synthesized data sets were generated with GeneNetWeaver^125^ for *E. coli* from the ground truth network of RegulonDB.^162^ The performance of each method was evaluated based on network analysis and prediction metrics such as ROC, AUC and PR, which highly varied across different data sets. Moreover, it was found that very few common sets of edges are detected by different methods, but a large number of method-specific and data-specific edges are observed for both experimental data and simulation data. Although this study has important value as being a pioneer in this field, it had the drawback of limited size of the input data sets in terms of number of genes. This study also suggests the importance of large sample sizes for data generation to derive the possible most accurate network inference. Moreover, the difference between data distribution of experimental data and simulated data affects the evaluation, even after imitating the real data by inducing dropout noise.

In addition, Nguyen et al^54^ conducted a comprehensive benchmarking on 15 GRN inference methods. These methods use diverse techniques to infer a network, such as Boolean models (BTR,^30^ SCNS,^121^ and Boolean Pseudotime Inference^163^), differential equation (inference snapshot,^32^ SCODE,^31^ and SCOUP^34^), expression correlation (empirical Bayes, information measures,^164^ SINCERA,^165^ NLNET,^40^ and SCENIC^28^), correlation based on pseudotime ordering (SCINGE,^119^ SCIMITAR,^36^ SINCERETIES,^35^ and LEAP^38^). The major aim of the study was to evaluate these methods by introducing technical variations in the data, such as the number of genes/sparsity. To achieve this goal, a total of 139 simulated data sets were generated using GeneNetWeaver^125^ based on known reference networks from the TRRUST^84^ database. All methods were evaluated for their accuracy in reconstructing reference networks, sensitivity to dropout rate and sparsity, and time complexity. The inconsistency in networks from different data sets shows that current methods are sensitive to technical noise and need to be more sophisticated to cope with the complex nature of the regulatory network from single-cell data. Moreover, many methods were designed to work with small-scale inputs and unable to cope with the rapidly increasing number of cells generated in single-cell data.

Similarly, Kang et al^81^ studied the stability of inferred networks with respect to technical variations in the input data, such as number of profiled cells, sequencing platform, and cell type annotation. This benchmarking was performed on 6 single-cell network inference methods based on their reproducibility in terms of their ability to infer similar networks when applied to 2 independent data sets for the same biological condition. The real data from 3 biological conditions: human retina, T-cells in colorectal cancer, and human hematopoiesis were considered to evaluate on highly different biological contexts. The number of genes in each data set was significant enough (>6000) for benchmarking. The inferred networks from each method and data set were evaluated using network comparison metrics such as perINT, WJS, RcisTarget score, and RegulatoryCircuit scores. The evaluation showed that for high link numbers (n: 100 000), GENIE3^6^ consistently generated the most reproducible results across all the 3 biological contexts considered. Furthermore, its performance proved to be stable with respect to the single-cell sequencing platform, the cell type annotation system and the number of cells considered. A rigid filtering (n: 1000 or 100), showed that CLR^4^ and GRNBoost2^117^ had better performance. However, even the best-performing methods show reproducibility scored less than ideal (26%-54% perINT and 0.1-0.3 WJS), indicating that further improvements are still needed in network inference methods for scRNA-seq data.

In one of the seminal studies in this field, Pratapa et al^80^ developed a benchmarking framework, BEELINE, and tested 12 diverse GRN inference algorithms on diverse data sets (synthesized, model generated, and experimental). The synthesized data sets were generated based on toy networks of different trajectories in Dynverse^128^ using the BoolODE^80^ method. Four published Boolean models of GRNs, reported for tissue differentiation and development, were used as these models reflect the real “ground-truth” control systems in biology. The selected models were for multicellular eukaryotes cortical area development (mCAD), ventral spinal cord (VSC) development, hematopoietic stem cell (HSC) differentiation, and gonadal sex determination (GSD). Five different single-cell RNA-seq data sets, 3 in mouse (mHSCs,^132^ mESC,^135^ and Mouse dendritic cells^136^) and 2 in human (hHEPs^138^ and hESCs^137^) containing a total of 7 cell types across these data sets. The data sets were processed through a uniform pipeline using different ground truth networks such as cell-type-specific ChIP-seq (ENCODE,^133^ ChIP-Atlas,^88^ and ESCAPE^82^ databases), nonspecific ChIP-seq (DoRothEA,^94^ RegNetwork,^91^ and TRRUST^84^) and functional interaction networks (STRINGdb^166^). This study evaluated the 8 algorithms that require pseudotime-ordered cells, such as data from cell differentiation and development processes. The performance evaluation was done based on AUPRC, EPR, stability across simulations (dropout or across algorithms), network motifs, software run time and memory usage PIDC,^39^ GENIE3,^6^ and GRNBoost2^117^ were the top performers. These methods consistently performed better for curated models and experimental data sets in terms of accuracy and better in recovering interactions in synthetic networks than Boolean models. The Boolean models with the best EPR performed better on experimental data sets as well and techniques that do not require pseudotime-ordering of the cells were found to be more accurate. Moreover, significant improvement in the EPR of the best-performing algorithms was observed by increasing the number of highly variable genes and considering all significantly variable TFs.

Moreover, to understand the suitability of a particular method for a specific research problem or experimental data, Zhao et al^69^ benchmarked 12 GRN methods. Methods were from diverse approaches such as Model-based (DBN,^167^ TIGRESS,^8^ and NonlinearODEs^168^), Information-based (CLR,^4^ ARACNe,^169^ PCA-CMI,^5^ CMI2NI,^170^ and PCA-PMI^171^), Machine learning-based (BiXGBoost,^172^ GENIE3,^28^ dynGENIE3,^173^ and JUMP3^174^) were tested on different data sets. The simulated data was taken from synthetic networks of DREAM4,^175^ real data from the RegulonDB database, and real data from published studies on SOS DNA repair (9 genes)^140^ and *E. coli* (1484 genes).^141^ The evaluation using AUPRC and AUROC showed that model-based methods TIGRESS^8^ and NonlinearODEs^168^ have better performance in constructing small-scale real networks (SOS DNA repair) compared with large-scale networks. However, these values on the *E. coli* data set differ significantly for each method because large-scale GRNs in biological systems are generally very sparse, which increases the difficulty of network inference. The machine learning-based methods generally yielded better results than model-based methods while inferring large-scale networks.

A recent expanded benchmarking study by McCalla et al uses diverse methods and gold-standard data sets. The study used 7 published scRNA-seq data sets from human,^148^ mouse,^136,149,150^ and yeast^73,74^ on 13 recent GRN inference methods. The evaluation was done using different gold standards (e.g. ChIP-chip/seq versus regulator perturbation) and their effect measured performance (Perturb, Chip-seq, Perturb + Chip). The methods were evaluated based on their computing requirements and their ability to recover the network structure and found that SCODE^31^ and SCENIC^28^ have better runtime and memory requirements. Some algorithms, such as SCHiRM,^176^ HurdleNormal,^177^ and BTR,^30^ did not complete in a reasonable amount of time and were excluded from downstream analyses. Furthermore, results of the assessment of the algorithms based on local network metrics AUPR, F-score, and PTFs suggest Pearson, SCENIC,^28^ MERLIN,^178^ and PIDC^39^ were consistent with the rankings across different gold standards. Evaluation based on recovery of network interactions showed that most of the methods did not recover major interactions; however, the master regulators of the system under study were recovered. Moreover, the imputation of scRNA-seq data sets did not improve network inference and addition of priors and TF activities was found to improve performance. Therefore, this study highlights the need for improved methods and better gold standards for regulatory network inference from scRNA-seq data sets.

Currently there is no true gold-standard reference; therefore, it is more difficult to identify a clear benchmark to assess performance for single-cell data. These benchmarking studies have only used input data generated from GeneNetWeaver and/or experimental data except BEELINE, where the synthetic data was generated in a GRN-guided manner. GeneNetWeaver is built for bulk data and as Chen and Mar^68^ reported for single-cell data, the network inference differs significantly. However, many GRN-guided synthetic data generation methods specifically designed for single-cell data have been made available recently. Some other studies used recent techniques for this purpose such as combining inference and simulation of GRNs.^179^ These tools can potentially facilitate more accurate benchmarking. Moreover, the input data size across benchmarking studies is not evaluated for stability and scalability for some benchmarking studies. We recommend the use of a sufficient size data set, in terms of number of cells and genes, as used in Kang et al^81^ for curated data.

Although the goal of benchmarking studies is to provide unbiased comparison of multiple methods, contrasting results across these studies raise additional questions. There are several potential reasons for this inconsistency. First reason is the usage of a diverse set of ground-truth networks for evaluating the accuracy (Table 5). The second reason is that the evaluation metrics reported in these benchmarking are also inconsistent. Employing standard metrics such as AUPRC and AUROC can address this problem. However, in some cases when the data set is highly imbalanced, AUPRC and AUROC might not be the best metrics for evaluating binary classifiers and other metrics such as EPR can be considered. Using different parameter settings may also cause discrepancy. Hence, either the methods should be run with the default parameters or the settings should be explicitly listed.

One common result from the aforementioned benchmarking studies is the low degree of accuracy for GRN inference from single-cell data for most methods. An alternative approach for increasing the accuracy is to use tissue-matching or multi-omic chromatin accessibility data. One such tool is LINGER^116^ (Lifelong neural network for gene regulation), which employs manifold regularization and integrates prior knowledge of TF motifs and atlas-scale external bulk data across many biological contexts. Other tools such as CellOracle^55^ and Pando^180^ also employ single-cell multi-omics data. These tools first use scATAC-Seq data and perform motif analysis to infer GRNs, which are further refined with a regression approach based on scRNA-Seq data. These 2 studies are also interesting in the sense that both of them report downstream experiments that proved the existence of predicted GRNs.

Currently, there is not any independent benchmarking study performed for single-cell multi-omic data and future studies can potentially fill this gap. Comparing multi-omics-based and expression-based methods is also an interesting topic, as the former group of methods exploits additional information. Normally having an extra data modality such as scATAC-Seq brings a natural advantage and we believe it should be exploited when available. However, this advantage may be lost when the quality of this additional data modality is low or even turn out to be a disadvantage depending on the characteristics of this data. An independent benchmarking comparing 2 groups of methods will help future investigators weigh the cost and benefits of using multi-omic data. Pseudotime-based methods are positioned similarly with the multi-omic data. Although they typically do not require additional data modality, the pseudotime values need to be computed in advance and they depend on both the accuracy of the pseudotime method and its parameter settings. The accuracy rates of these methods were different on synthetic, model generated, and experimental data sets. SINCERITIES^35^ performs best on synthetic data sets, however very poorly on experimental single-cell data sets. Similarly, SCODE^31^ performs best on some data sets when evaluated using ChIP-seq as ground truth. However, these methods are having scalability issues and need to be evaluated on the same pseudotime generation method such as Slingshot.^181^

High-throughput perturbation technologies present a new dimension of data for constructing GRNs. In this context, single-cell CRISPR screening protocols such as Perturb-seq^182^ and CROP-seq^183^ measure the gene expression under different genetic knockout/knockdown. This type of data set is promising as it can help with both GRN inference and benchmarking. In recent years, multiple studies came out that use CRISPR screening data for GRN inference and benchmarking such as McCalla et al,^70^ CausalBench,^184^ CellOracle,^55^ and Pando.^180^

## Discussion

Benchmarking GRN methods is far from a straightforward task due to the underlying complexities of gene regulation and a large variety of selections for gold-standard data sets. Different benchmarking studies have used various data sets, methods, ground truth networks and evaluation strategies. While some of these studies attempted to evaluate the performance of single-cell specific methods over bulk sequencing-based methods, others focused on methods addressing specific research problems such as differentiation process or a steady state. Due to this biological (input data) and technical (methods and evaluation metrics) heterogeneity, there are differences in the results of benchmarking studies.

In terms of input, to generate synthetic data sets, Gene NetWeaver^125^ has been widely used across benchmarking studies. However, due to its limitations for handling sparse data and pseudotime based data, BoolODE^80^ was developed in the BEELINE^80^ framework. Moreover, some other methods such as SERGIO and GRouNdGAN have been recently developed to overcome the challenges in single-cell data. These methods can handle the stochastic nature of single-cell data and are specifically useful in benchmarking pseudotime requiring methods for different network topologies and cell types. However, BEELINE directly used simulation times for datasets generated from toy networks only; for datasets generated from curated models, it used Slingshot for GRN inference.^181^

In terms of metrics, AUROC and AUPRC are the most used evaluation metrics. The AUROC provides an easy interpretation of performance, as 0.5 represents the random guess baseline. When the network is moderately dense or the class distribution (ie, the balance of positive and negative edges) is present, AUROC is favorable. It assesses a model’s capacity to discriminate between accurate and inaccurate positive predictions made at various threshold levels. This metric offers a comprehensive evaluation of the discriminative capability of the model and is resistant to class imbalance. However, because AUROC handles all forms of mistakes equally over the whole range of possible thresholds, it may not be able to accurately quantify performance in highly imbalanced networks or when the focus is on certain subsets of interactions. However, AUPRC can handle class imbalance better. In many real-world scenarios, including GRN analysis, the number of negative examples (non-interactions) often highly exceeds the number of positive examples (interactions). AUPRC is less affected by class imbalance as it focuses on the precision (PPV) of the model, making it particularly useful when the objective is to minimize false positives. Other accuracy metrics can be useful for specific problems. For example, EPR and F-score can provide insights into local interactions whereas PerInt and WJS are suitable for dense networks.^81,185^ In summary, the choice of evaluation metric should be guided by the specific characteristics of the GRN being analyzed, including scale, density, and directionality. Use of multiple metrics can provide a more comprehensive assessment of the model performance. In addition, it is essential to consider the biological context and relevance of the metrics to ensure meaningful interpretation of the results. The large difference in performance per data set used in these studies is majorly due to the fact that these studies use different ground truths for evaluation. Moreover, the parameter settings used for each method is not discussed in all studies.

The ground truth network used in different studies is an important aspect in the reliability of benchmarking. Each benchmarking study has used a different set of ground-truth networks, although some repositories such as RegulonDB^72^ and STRING^96^ have been used in multiple studies. Building a repository of ground-truth networks and associated input data is an important endeavor in this field. The BEELINE^125^ database is an important milestone in this context and additional repositories in this context can significantly contribute to standardization of benchmarking.

An important issue in benchmarking is accounting for indirect interactions as in a scenario where gene A regulates gene B, which also regulates gene C. It is of question whether the indirect interactions should be represented with edges as well. In our view, an ideal GRN should contain direct edges only as placing edges for indirect edges will lead to inaccurate representations. For example, for the scenario described above, placing an edge between A and C for indirect regulation may cause the reader to speculate that gene A will continue to regulate gene C even if gene B is knocked down, which is not true as this regulation is mediated through gene B. Moreover, indirect relationships can always be inferred based on the network topology from the direct relationships. Hence, we believe that ideally, the GRNs must solely be composed of direct interactions.

It is further to note that the traditional GRN inference algorithms are mainly based on gene expression data, but new GRN inference methods have been proposed that use single-cell multi-omics data sets, and there are also methods emerging that infer cell-level GRNs. Recent GRN inference methods leverage supplementary modalities such as TF using ChIP-seq and ATAC-seq, DNA methylation and additional information, such as pseudotime. Data availability for different modality is increasing and there is no benchmarking study available for single-cell multi-omics-based GRN construction methods. A potential benchmarking approach can be using paired scRNA-seq and enhancer information predicted with epigenomic data. Synthetic data can be also generated for such data types using existing simulation based tools such as scDesign3^159^ and scMultiSim.^160^ Integration of 2 modalities can improve the true positive rate in inferred GRN. This study also suggests benchmarking of such methods alongside methods only inputting gene expression.

## Conclusion

There has been a considerable amount of research on building GRNs in recent decades. Due to its importance in understanding disease-related pathways and thanks to the fast pace of advances in genomics technologies, especially in single-cell multi-omics, interest in regulatory networks is only expected to increase. Hence, we anticipate that novel methodologies will be proposed for building GRNs in the future.

This review presented a brief overview of the current approaches for benchmarking GRNs, together with their strengths and limitations, and highlighted potential ways of addressing these limitations. The presented knowledge will guide future investigators in establishing benchmarking approaches to assess the accuracy of GRN methods and to develop more accurate and usable tools.
